# Association of Post-streptococcal Glomerulonephritis and Paediatric Posterior Reversible Encephalopathy Syndrome (PRES): A Case Report

**DOI:** 10.7759/cureus.98043

**Published:** 2025-11-28

**Authors:** Pawan K Ghanghoriya, Ranjith Kumar V, Ashik Ali M S, Jayas Jagan, Shaunak Rangarh

**Affiliations:** 1 Department of Paediatrics, Netaji Subhash Chandra Bose Medical College, Jabalpur, IND

**Keywords:** haematuria, hypertensive emergencies, paediatric neuroimaging, posterior reversible encephalopathy syndrome, post-streptococcal glomerulonephritis

## Abstract

Posterior reversible encephalopathy syndrome (PRES) is a rare clinical entity with diverse etiological associations and unclear pathophysiological mechanisms. We report a case of post-streptococcal glomerulonephritis (PSGN) complicated by PRES, characterized by a classic clinical course and distinctive neuroimaging findings. A 13-year-old boy presented with complaints of headache, vomiting, blurred vision, and multiple generalized seizure episodes with post-ictal confusion. He had a history of sore throat and fever 14 days back, followed by abdominal pain, generalized oedema, and persistent frank haematuria. On admission, he had hypertension (blood pressure above the 99th percentile for age). Contrast-enhanced CT head revealed hypodensities in white matter (centrum semiovale extending to posterior parietal and occipital lobes) suggestive of vasogenic oedema. Follow-up MRI brain after two weeks of admission demonstrated resolution of oedema. Given the clinical context and imaging findings, a diagnosis of PSGN complicated by PRES was made. PSGN is the most common cause of acute, severe hypertension in the paediatric age group. This case highlights the association between PSGN and PRES and underscores the importance of prompt recognition and management of hypertension to ensure favourable outcomes.

## Introduction

Posterior reversible encephalopathy syndrome (PRES) is a rare, acute neurological condition characterized by a spectrum of neurological and radiological features, with various underlying aetiologies. First described by Hinchey et al. in 1996, PRES has been documented in numerous case reports since then [1]. Clinical manifestations of PRES include seizures, altered consciousness, headache, and visual disturbances (e.g., blurring of vision, reduced visual acuity, and disturbances in colour vision). Risk factors commonly associated with PRES encompass hypertension, sepsis, renal parenchymal disease, immunosuppressive drugs, and autoimmune disorders [2,3]. Notably, renal insufficiency, followed by haematological disorders and related therapies, and the use of immunosuppressive medications, have been identified as the most common triggering factors for PRES in the paediatric population [3].

A nationwide study indicates that the mean age of PRES onset in children is around 12.5 years, with a slight female preponderance. Recent studies estimate the incidence of PRES in the general paediatric population to be approximately 0.04%, with higher rates observed among children undergoing cancer treatment (0.7%) and those admitted to intensive care units (0.7%) [4]. The prevailing pathophysiological theory is the vasogenic hypothesis, which posits that a sudden elevation in blood pressure leads to a failure in cerebral autoregulation, resulting in disruption of the blood-brain barrier. This disruption is particularly evident in the posterior circulation, including the basilar and vertebrobasilar regions, due to their reduced sympathetic innervation. In recent years, PRES has been widely reported with global application of neuroimaging techniques [5-8].

Here, we present a compelling case of a 13-year-old boy who was admitted with complaints of headache, multiple episodes of projectile vomiting, and subsequent seizures. He was diagnosed with post-streptococcal glomerulonephritis (PSGN) complicated by acute severe hypertension, leading to the development of PRES.

## Case presentation

A 13-year-old boy was referred to our tertiary care centre with complaints of multiple episodes of abnormal body movements (tonic-clonic jerks), each lasting for 5-10 minutes, with unresponsiveness. There were a total of four episodes over four hours, and the child was conscious in between episodes.

Detailed history revealed that the child had a history of fever and sore throat two weeks prior, which resolved spontaneously within two to three days. After seven days of fever, he developed facial puffiness (day one of illness). By day three of illness, swelling gradually progressed to the whole body. He also noted painless dark brown urine, which was persistent and not associated with urgency or hesitancy. The child was admitted to a nearby district hospital for further evaluation on day five of illness. On the eighth day of illness, he experienced a dull headache upon awakening, followed by multiple episodes of projectile vomiting and blurring of vision. Later that evening, he had four episodes of generalized tonic-clonic seizures (GTCS), each lasting approximately 5-10 minutes. There was no history of rash or skin lesions, joint pain, focal motor deficits, vomiting, jaundice, or bleeding from any site other than haematuria. There was no history of drug intake. He was treated with intravenous antiepileptics and subsequently transferred to our centre for further management.

The child had normal development, was studying well in the seventh standard, and was immunized as per the National Immunization Schedule of India. He had no significant past medical history.

On arrival to the emergency triage, he was irritable, tachycardic (heart rate - 124/min), hypertensive (blood pressure - 196/122 mmHg (>99th percentile for age), and not following commands (Glasgow Coma Scale (GCS) score of 14/15). His respiratory rate was 24/min, and SpO₂ was 96% on room air. Neurological examination was unremarkable, except for decreased vision in both eyes. Other general and systemic examinations were within normal limits.

Differential diagnoses considered were metabolic encephalopathies, namely, hyponatremia, hypernatremia, hypocalcaemia, uremic encephalopathy, hypertensive encephalopathy, PRES, collagen vascular disorders, and intracranial bleed.

**Figure 1:**
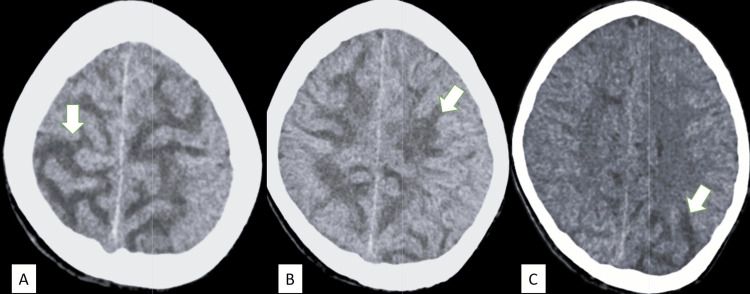
(A&B): CT head axial section at the centrum semiovale level showing bilateral symmetric hypodensities in white matter with persevered white-grey differentiation (arrows). (C): Section showing bilateral asymmetrical white matter hypodensities in the posterior parietal lobe (arrows), suggestive of vasogenic oedema.

Contrast-enhanced CT (CECT) of the head revealed white matter hypodensities in bilateral centrum semiovale extending to posterior parieto-occipital regions, suggestive of vasogenic edema. No haemorrhage was noticed (Figure 1). Bedside abdominal ultrasonography showed mildly increased renal cortical echogenicity. Electrocardiography showed sinus tachycardia. Laboratory investigations revealed non-nephrotic range proteinuria (<40 mg/m² BSA/day), elevated anti-streptolysin O (ASO) titer (>200 IU), uremia (69.59 mg/dL), low serum complement C3 (11.2 mg/dL), and frank haematuria on urine microscopy (Table 1). Echocardiography and chest X-ray were unremarkable.

**Table 1:** Laboratory investigations. ALT: alanine transaminase; ALP: alkaline phosphatase; AST: aspartate aminotransferase; ANA: anti-nuclear antibody; C-ANCA: cytoplasmic anti-neutrophil cytoplasmic antibody; ESR: erythrocyte sedimentation rate; HDL: high-density lipoprotein; LDL: low-density lipoprotein; P-ANCA: perinuclear anti-neutrophil cytoplasmic antibody

Laboratory Investigations	Day 1	Normal Range
Hb (g/dL)	8.8	12.4-16.4
Total WBC count (µL)	8.69	4.8-10.8
Packed cell volume (%)	26.90	30-44
Mean corpuscular volume (fL)	72.4	80-96
Mean corpuscular hemoglobin (pg)	23.7	27-31
Platelet count (µL)	440,000.0	150,000-450,000
Blood urea (mg/dL)	69.59	7-20
Serum creatinine (mg/dL)	1.55	0.45-0.81
Total bilirubin (mg/dL)	0.29	0.1-1.2
Direct bilirubin (mg/dL)	0.05	<0.3
Total protein (g/dL)	6.15	6-8.3
Albumin (mg/dL)	3.05	3.4-5.4
Uric acid (mg/dL)	10.12	3.5-7.2
AST (IU/L)	40.3	10-40
ALT (IU/L)	27.10	10-40
ALP (IU/L)	187.20	<350
Globulin (g/dL)	2.2	2-3.5
Triglycerides (mg/dL)	42.64	0-150
Total cholesterol (mg/dL)	121.07	40-200
HDL-cholesterol (mg/dL)	31.25	35.3-79.5
LDL-cholesterol (mg/dL)	81.00	0-130
Serum sodium (mEq/L)	138.3	135-145
Serum potassium (mEq/L)	4.17	3.4-4.7
Serum chloride (mEq/L)	100.7	98-106
Serum calcium (mg/dL)	7.62	9-10.5
E.S.R (mm/hour)	40	0-20
Activated partial thromboplastin time(seconds)	50.50	27-42.5
Prothrombin time test (seconds)	16.90	11-16.5
Prothrombin time INR	1.30	0.70-1.20
Urine color	Reddish	Pale yellow
Protein	+	Absent
RBCs	Full field/HPF	Not seen
Pus cells	4-5/HPF	0-2
Antistreptolysin O titres(IU/mL)	>200	<200
ANA (AU/mL)	17.8	Negative
Anti-ds DNA(<25 IU/mL)	6	Normal
C-ANCA(<19 AU/ML)	0	Normal
P-ANCA(<19 AU/ML)	3	Normal
Serum complement 3 (C3) levels (mg/dL)	11.2	80-150

Based on the clinical presentation of acute nephritic syndrome (edema, hypertension, and hematuria) with raised ASO titer diagnosis of PSGN was considered, with characteristic neuroimaging findings suggestive of PRES. The child was transferred to the Paediatric Intensive Care Unit (PICU) for further management.

An intravenous labetalol infusion was initiated, and desired blood pressure reduction targets were calculated. The goal was a 25% reduction in blood pressure over the first eight hours, followed by gradual normalization over the next 24-48 hours. However, blood pressure reduction was not achieved within the expected timeframe despite optimal labetalol dosing. Therefore, oral angiotensin-converting enzyme (ACE) inhibitors and calcium channel blockers were added after 24 hours of treatment, alongside ongoing labetalol infusion. Over the next 48 hours, blood pressure gradually normalized to below the 90th percentile for age. Labetalol was tapered and stopped after 48 hours. Oral anti-hypertensives were continued.

**Figure 2:**
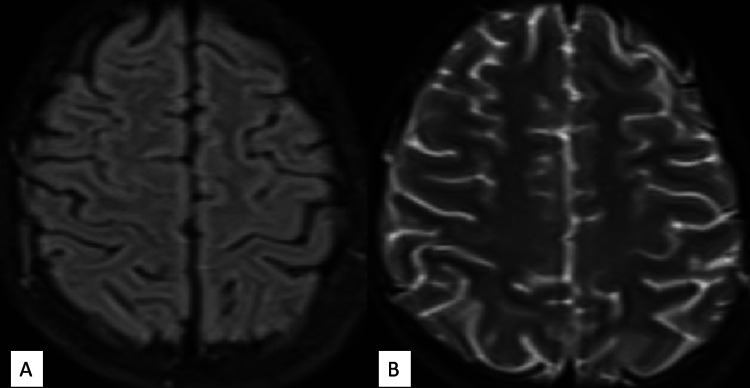
(A&B): Follow-up MRI showing the disappearance of oedema, characteristic of posterior reversible encephalopathy syndrome.

The child became fully conscious and following commands after six hours of admission, and by the fourth day of admission, vision became normal (visual acuity 6/6), and no further seizures, headaches, or vomiting were reported. By the 10th day of hospitalization, oral antihypertensive medications were tapered and eventually discontinued. The child was discharged with recommendations for weekly follow-up, a sodium-restricted diet, and repeat neuroimaging. Follow-up MRI brain after seven days (17 days after CECT head) revealed complete resolution of previous abnormalities, confirming the diagnosis of PRES (Figure 2).

The child remained clinically stable on subsequent follow-ups and off drugs for three months.

## Discussion

Acute glomerulonephritis (AGN) is characterized by variable degrees of hematuria, edema, hypertension, and oliguria. β-hemolytic streptococcal infection is the most common cause of acute nephritic syndrome in children. Most of the children have a sore throat or pyoderma, followed by AGN after a latent period of 7-14 days. Diagnosis is confirmed by high ASO titer, low C3 levels, and reversibility of the condition. Some cases may have neurological complications such as hypertensive encephalopathy/PRES, uremic encephalopathy, or acute disseminated encephalomyelitis (ADEM). Our case presented with classical features of PSGN complicated by PRES.

PRES is an acute neurologic syndrome with various clinical manifestations and characteristic radiological features. Although it is more commonly reported in adults, recent case studies suggest an increasing incidence in paediatric populations. Despite the “reversible” nature often attributed to its radiological findings, PRES has been reported to have 3.2% mortality in the paediatric population and an overall mortality of around 16% across different age groups [3,9,10]. Clinically, paediatric PRES is characterized by a seizure, altered consciousness, headache, impaired vision, and focal neurological deficits and characteristic radiological findings. Our case had all the classical clinical features of PRES. Notably, the paediatric brain differs from the adult brain in several aspects, including autoregulatory capacity, susceptibility to noxious stimuli, and sympathetic innervation, which may explain why children often exhibit somewhat different clinical features and atypical radiological findings compared with adults [11].

A wide age range (4-90 years) has been noted in reported cases, with a mean age of 44.2 years, and a female preponderance suggesting possible gender‐based susceptibility [12]. Paediatric PRES is frequently reported in children having hypertension, autoimmune disorders, renal disorders, and those receiving chemotherapy or immunosuppressive medications. Often, childhood hypertension is overlooked in the context of seizure episodes, where elevated intracranial pressure (ICP), agitation due to the seizure episode, are thought to be causing a transient rise in blood pressure. In such cases, hypertension may be misattributed, rather than recognised as a causative factor in acute encephalopathy and seizure presentation. Our case had PSGN with severe hypertension.

Compared to adults, children are more susceptible to developing PRES in association with severe hypertension, likely due to their lower thresholds for maintaining cerebral autoregulation. In some reports, mean arterial pressure thresholds in children are cited as low as ~40 mmHg, compared with ~60 mmHg in adults. Therefore, PRES might be more common in children than previously recognized, though possibly underdiagnosed [13]. In one large review, 88% of children who developed PRES in oncological settings had hypertension [14], and 80% of cases had documented hypertension above the 99th percentile for at least six hours before symptom onset. These findings emphasize that aggressive management of hypertension could prevent progression to cerebral edema, irreversible neurological injury, and poor long‐term neurodevelopmental outcomes.

Typical radiological finding is the presence of vasogenic edema in the parieto-occipital cortex, but it may affect frontal and temporal lobes as well, extending from subcortical white matter to the periventricular white matter according to the severity. Parieto-occipital pattern is classic, but other common patterns are the holohemishpheric watershed pattern and superior frontal sulcus pattern. A meta-analysis reported near equal incidences of parieto-occipital pattern and holohemispheric pattern that suggest ‘posterior’ in PRES is a misnomer [15]. PRES can be complicated by hemorrhage in 15-65% cases and by cytotoxic edema in 11-26% of cases, indicating irreversibility and poor outcome in these cases [16]. Our case had an atypical pattern of holohemispheric involvement without any hemorrhage and complete reversal of lesions.

The pathophysiological mechanism underlying PRES remains under debate. The most widely accepted theory is the vasogenic theory, which postulates that acute, severe hypertension leads to endothelial injury and impairment of cerebral autoregulation. This ultimately results in fluid exudation into the interstitium across the blood-brain barrier. Distribution of vasogenic edema in the watershed area suggests that hypoperfusion may play a role secondary to exaggerated vasoconstriction. Although this theory is compelling, multiple cases have been reported in patients who are normotensive. To account for such cases, the cytotoxic (endothelial dysfunction) theory is invoked: endothelial injury secondary to immunosuppressants or cytotoxic drugs, autoimmune disorders, or fluctuations in blood pressure may damage the cerebral endothelium, leading to vasoconstriction, dysregulated autoregulation, and subsequent oedema. More recently, hypotheses involving dysregulated vascular endothelial growth factor (VEGF) secretion [17] and upregulation of arginine vasopressor receptors in cerebral circulation have been proposed [18], which may guide future therapeutic strategies.

Differentiation of PRES from other acute neurological entities is crucial, since management strategies differ, and some supportive measures used for other conditions (e.g., aggressive maintenance of cerebral perfusion pressure) might be contraindicated in PRES. In such cases, a thorough history and serial neuroimaging, with early suspicion of PRES, are key to ensuring good outcomes.

Symptom‐directed management is imperative in children who develop PRES: anticonvulsants for seizure control, graded reduction of hypertension, and removal of offending medications and triggering factors. Although many studies show good outcomes, complications do occur, such as cerebral haemorrhage, ischemic stroke, and recurrence of PRES [19]. Factors associated with poorer prognosis in paediatric PRES include the presence of raised inflammatory markers, admission to intensive care (PICU), status epilepticus at presentation, and atypical MRI lesions [20].

## Conclusions

This case highlights a rare but important neurological complication of PSGN in children. PRES should be considered in patients presenting with hypertensive encephalopathy, seizures, and visual disturbances, especially in the context of recent streptococcal infection. PRES is a neuroradiological diagnosis with characteristic neuroimage findings of parietooccipital vasogenic edema, but other atypical patterns are also common. Early recognition and aggressive blood pressure management are essential for favourable outcomes.

## References

[1] Hinchey J, Chaves C, Appignani B (1996). A reversible posterior leukoencephalopathy syndrome. N Engl J Med.

[2] McCoy B, King M, Gill D, Twomey E (2011). Childhood posterior reversible encephalopathy syndrome. Eur J Paediatr Neurol.

[3] Thavamani A, Umapathi KK, Puliyel M, Super D, Allareddy V, Ghori A (2020). Epidemiology, comorbidities, and outcomes of posterior reversible encephalopathy syndrome in children in the United States. Pediatr Neurol.

[4] Raj S, Overby P, Erdfarb A, Ushay HM (2013). Posterior reversible encephalopathy syndrome: incidence and associated factors in a pediatric critical care population. Pediatr Neurol.

[5] Gao B, Lyu C, Lerner A, McKinney AM (2018). Controversy of posterior reversible encephalopathy syndrome: what have we learnt in the last 20 years?. J Neurol Neurosurg Psychiatry.

[6] Tetsuka S, Ogawa T (2019). Posterior reversible encephalopathy syndrome: a review with emphasis on neuroimaging characteristics. J Neurol Sci.

[7] Donmez FY, Basaran C, Kayahan Ulu EM, Yildirim M, Coskun M (2010). MRI features of posterior reversible encephalopathy syndrome in 33 patients. J Neuroimaging.

[8] Bartynski WS (2008). Posterior reversible encephalopathy syndrome, part 2: controversies surrounding pathophysiology of vasogenic edema. AJNR Am J Neuroradiol.

[9] Legriel S, Schraub O, Azoulay E (2012). Determinants of recovery from severe posterior reversible encephalopathy syndrome. PLoS One.

[10] Alhilali LM, Reynolds AR, Fakhran S (2014). A multi-disciplinary model of risk factors for fatal outcome in posterior reversible encephalopathy syndrome. J Neurol Sci.

[11] Siebert E, Bohner G, Endres M, Liman TG (2014). Clinical and radiological spectrum of posterior reversible encephalopathy syndrome: does age make a difference? - A retrospective comparison between adult and pediatric patients. PLoS One.

[12] Lee VH, Wijdicks EF, Manno EM, Rabinstein AA (2008). Clinical spectrum of reversible posterior leukoencephalopathy syndrome. Arch Neurol.

[13] Brady TM, Solomon BS, Neu AM, Siberry GK, Parekh RS (2010). Patient-, provider-, and clinic-level predictors of unrecognized elevated blood pressure in children. Pediatrics.

[14] de Laat P, Te Winkel ML, Devos AS, Catsman-Berrevoets CE, Pieters R, van den Heuvel-Eibrink MM (2011). Posterior reversible encephalopathy syndrome in childhood cancer. Ann Oncol.

[15] Orlando C, Milani GP, Simonetti GD (2022). Posterior reversible leukoencephalopathy syndrome associated with acute postinfectious glomerulonephritis: systematic review. Pediatr Nephrol.

[16] Brady E, Parikh NS, Navi BB, Gupta A, Schweitzer AD (2018). The imaging spectrum of posterior reversible encephalopathy syndrome: a pictorial review. Clin Imaging.

[17] Marra A, Vargas M, Striano P, Del Guercio L, Buonanno P, Servillo G (2014). Posterior reversible encephalopathy syndrome: the endothelial hypotheses. Med Hypotheses.

[18] Largeau B, Le Tilly O, Sautenet B, Salmon Gandonnière C, Barin-Le Guellec C, Ehrmann S (2019). Arginine vasopressin and posterior reversible encephalopathy syndrome pathophysiology: the missing link?. Mol Neurobiol.

[19] Hinduja A (2020). Posterior reversible encephalopathy syndrome: clinical features and outcome. Front Neurol.

[20] Komur M, Ozgur A, Delibas A, Bozlu G, Alakaya M, Direk M, Okuyaz C (2021). Posterior reversible encephalopathy syndrome (PRES) due to acute hypertension in children: 12 years single-center experience. Acta Neurol Belg.

